# Idiopathic Intracranial Hypertension Masking as Obstructive Hydrocephalus: A Case Report

**DOI:** 10.7759/cureus.4420

**Published:** 2019-04-09

**Authors:** Robert Maurer, April Henry, Kenneth C Liu, Brad Zacharia

**Affiliations:** 1 Neurosurgery, Penn State Milton S. Hershey Medical Center, Hershey, USA

**Keywords:** idiopathic intracranial hypertension, pineal gland cyst, headaches, pseudotumor cerebri, obstructive hydrocephalus, venous sinus stenting

## Abstract

Idiopathic intracranial hypertension (IIH) is a poorly understood phenomenon and its presentation can both mimic and co-exist with other intra-cranial processes. Accurate diagnosis is imperative as ongoing advancements in treatment can yield dramatic positive results. Here we present the case of an individual with signs and symptoms of obstructive hydrocephalus who was ultimately found to have IIH secondary to venous sinus stenosis. After correction of the venous sinus stenosis, resolution in the patient's symptoms was noted. The case highlights some of the unique considerations in approaching patients with IIH and provides a framework for review of current literature related to IIH and venous sinus stenosis.

## Introduction

Idiopathic intracranial hypertension (IIH) is a mysterious pathologic process wherein intracranial pressure (ICP) is elevated without a clear source. It is possible that other intracranial processes can present concurrently with IIH which can make an accurate diagnosis challenging. We detail the case of a patient with an unusual presentation of IIH which, once accurately diagnosed and promptly treated, responded with outstanding results.

## Case presentation

A 28-year-old woman initially presented with blurry vision that developed over the span of approximately one month. The blurry vision was initially most prevalent on horizontal gaze but progressed to include vertical gaze. It resolved with closure of one eye. She reported a history of gradually worsening headache over the past several years. Her headaches both worsened in intensity and increased in frequency, until it was quite debilitating and occurred daily. She described the headache as an intense pressure in both the front and back of her head. She also noted a “whooshing” sound in her right ear. She denied any nausea or vomiting and had not had any syncope, numbness, weakness, facial droop or slurred speech. Furthermore, she had no history of bladder or bowel dysfunction.

Her medical history was pertinent only for obesity with a body mass index (BMI) of 39. On physical exam she was noted to have papilledema. Her neurological exam was unrevealing with the exception of a subtle sixth cranial palsy.

**Figure 1:**
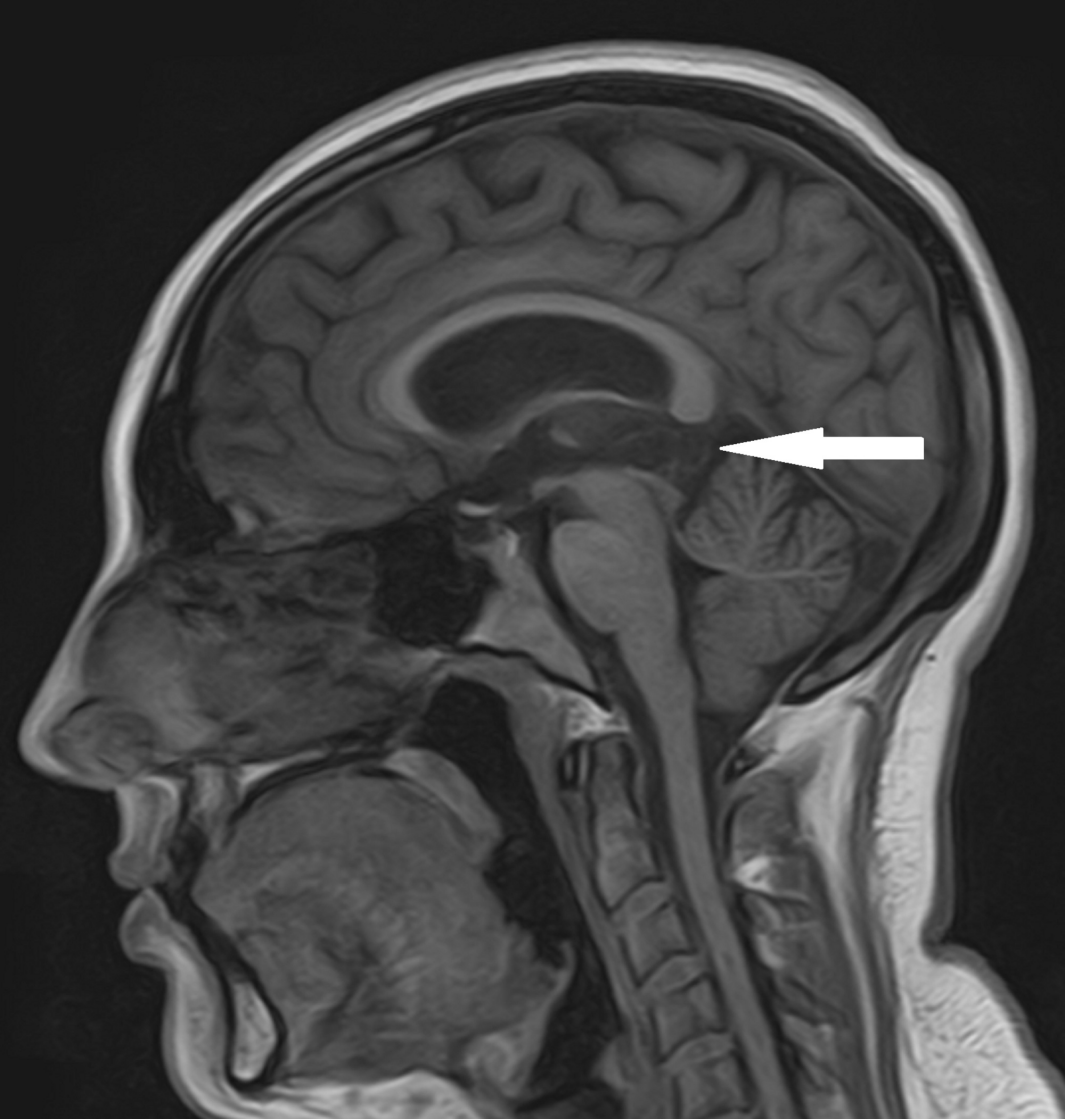
T1-weighted non-enhanced sagittal magnetic resonance imaging (MRI) demonstrating compression of the tectum by the cyst (highlighted by the white arrow).

**Figure 2:**
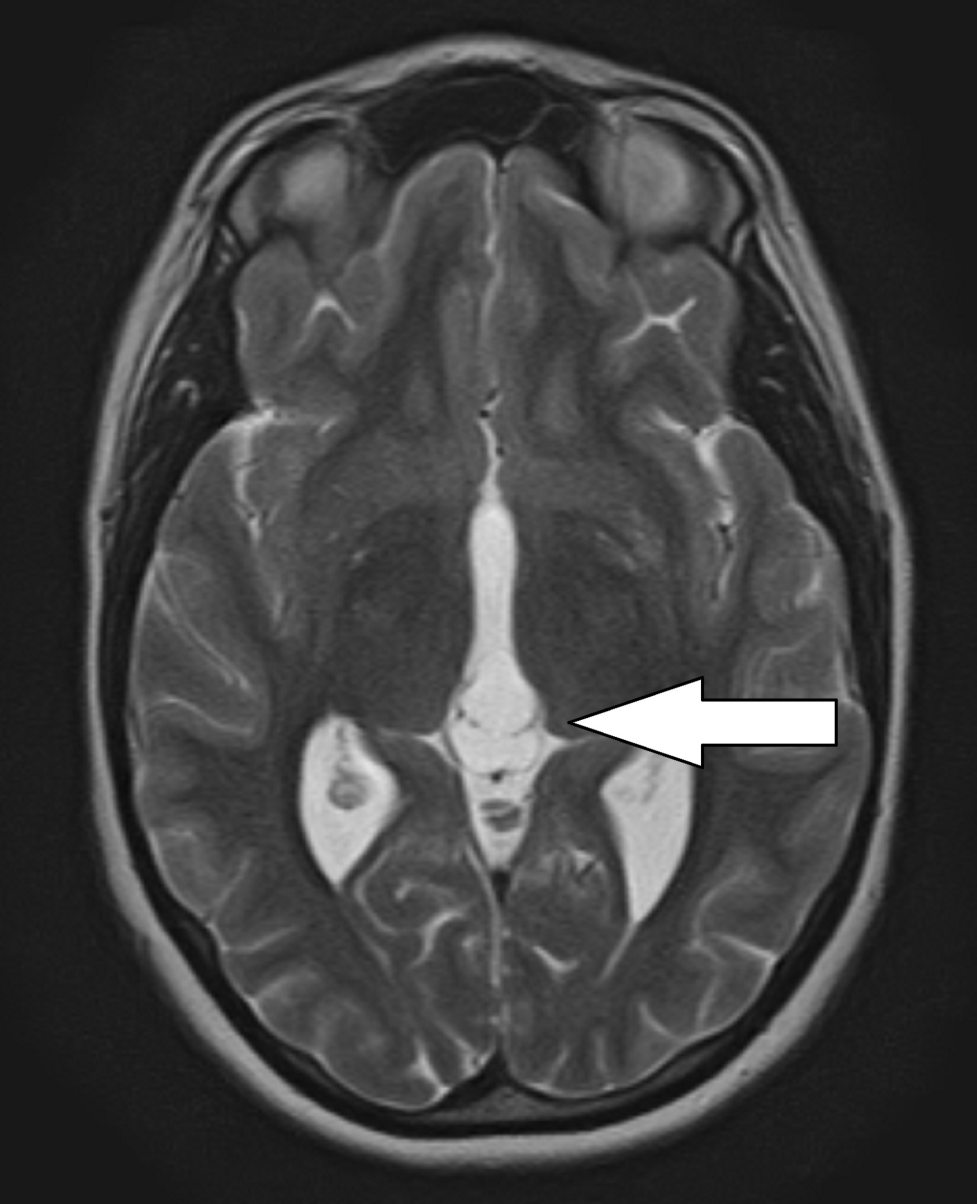
T2-weighted axial magnetic resonance imaging (MRI) demonstrating septated pineal cyst.

A magnetic resonance image (MRI) was obtained which showed a T1 hypointense and T2 hyperintense cystic lesion arising from the pineal gland measuring 2.0 x 1.1 cm in the sagittal plane with mild mass effect on the tectum and partial effacement of the cerebral aqueduct (Figures 1, 2). The lesion demonstrated a thin rind of contrast enhancement and had thin enhancing internal septations. The lateral ventricles were mildly enlarged. There was no restricted diffusion and no loss of gray white differentiation. Cine flow study noted cerebral spinal fluid (CSF) flow through the cerebral aqueduct. Based on the radiographic images, the most likely diagnosis was an atypical pineal cyst.

**Figure 3:**
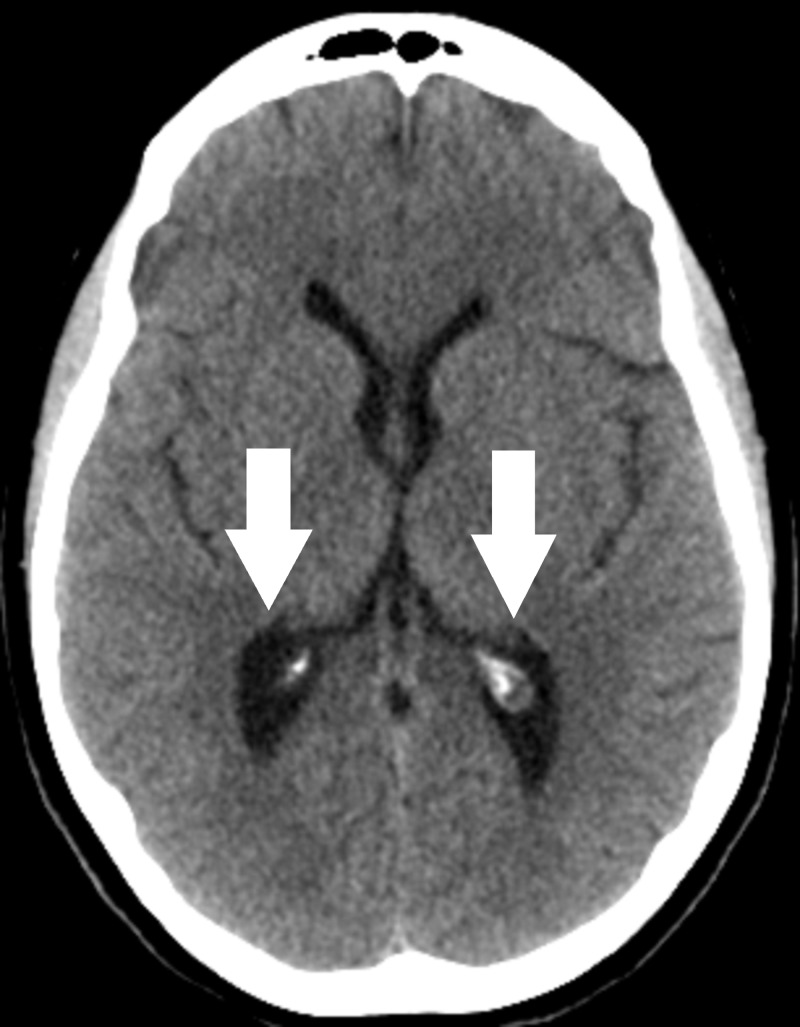
Axial computed tomography (CT) following endoscopic third ventriculostomy. Note unchanged size of lateral ventricles.

**Figure 4:**
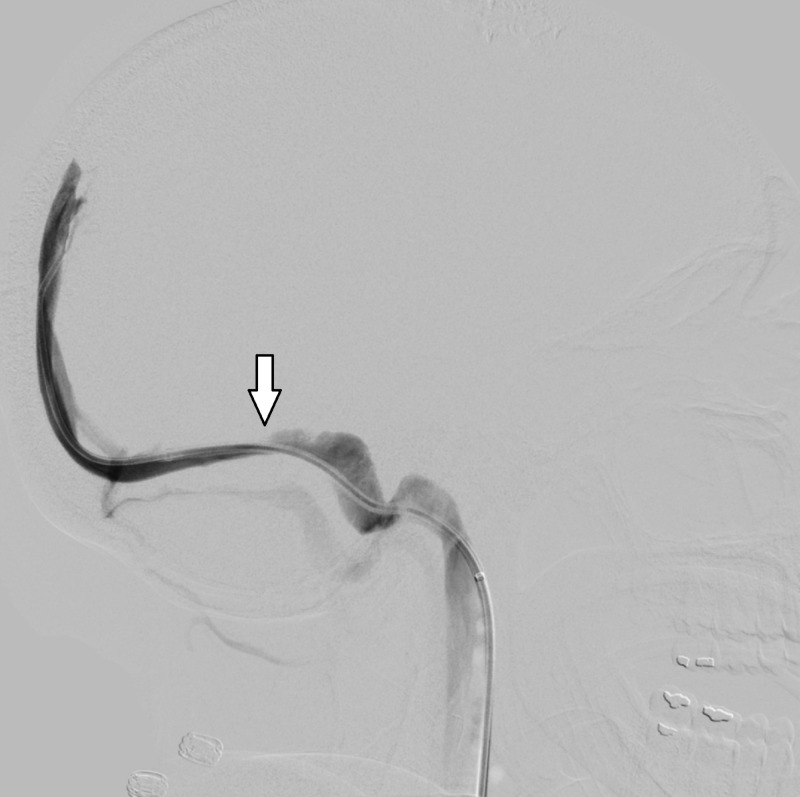
Lateral catheter angiogram demonstrating focal stenosis in left transverse sinus.

**Figure 5:**
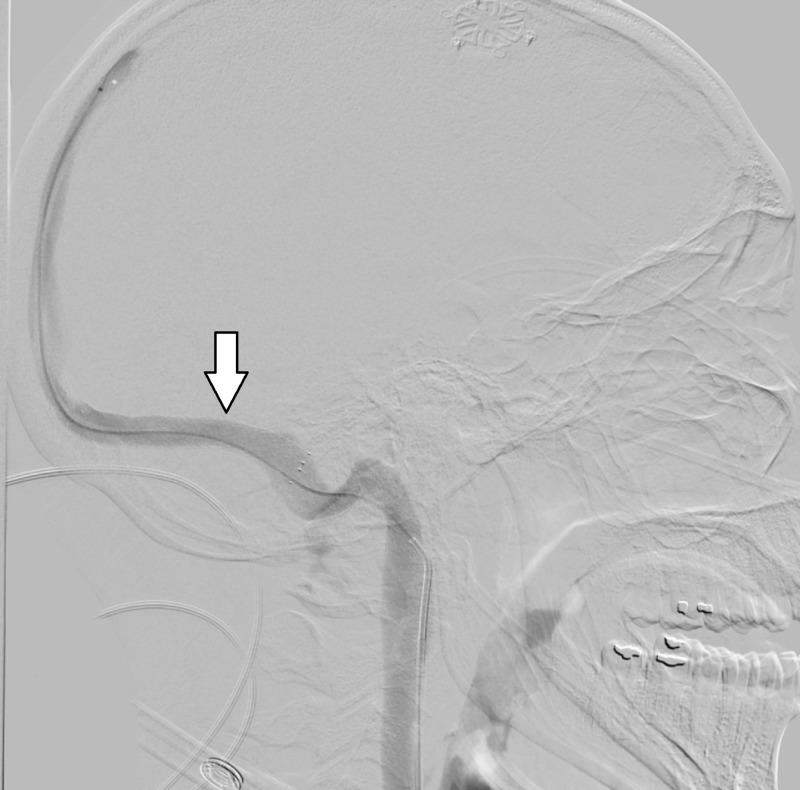
Lateral catheter angiogram demonstrating resolution of the focal stenosis following placement of a transverse sinus stent.

Given the rapidity of the vision changes, the decision was made to pursue surgical intervention. An endoscopic third ventriculostomy (ETV) with pineal cyst fenestration was performed without complication. A computed tomography (CT) scan obtained post-operatively noted questionable decompression of the lateral ventricles but the patient reported no improvement in symptoms (Figure 3). Ophthalmologic evaluation noted worsened papilledema. At this time the patient underwent a lumbar puncture, which noted an opening pressure of 32 cm H2O. Subsequent catheter venography noted severe stenosis of the right transverse sinus associated with a 9 mm Hg trans-stenosis gradient (Figure 4). Placement of a venous sinus stent obliterated the pressure gradient (Figure 5).

At six-month follow-up, her blurred vision, headaches and papilledema had all resolved. She reported complete resolution of her symptoms and plans were made for continued annual follow-up to monitor symptoms and ensure patency of the stent.

## Discussion

Intracranial hypertension is an abnormal increase in the CSF greater than 25 cm H2O. While this can be a result of brain tumors, certain endocrine disorders, prior infection and venous thrombi in hypercoagulable states, there are a number of cases that have no discernable cause. The first case of IIH was described in 1897, however, it was not formally recognized clinically until the 1940s [1]. This disorder was originally described as benign intracranial hypertension in 1955, however, this name was later changed to pseudotumor cerebri and ultimately is now referred to as idiopathic intracranial hypertension. These changes in nomenclature reflect an effort to adequately represent the clinical implications of the disorder which can include severe vision loss with papilledema [2].

The classic presentation of IIH is an obese woman of childbearing age presenting with a severe headache and papilledema. It is unclear exactly why obesity is so closely related to the development of IIH, however, it is thought that the increased intra-abdominal pressure could lead to an increase in the filling pressure of the right heart, resulting in increased venous pressure [3]. While the exact pathogenesis of IIH is not known, the prevailing theory focuses on impaired CSF resorption [1,4]. Another explanation is increased cerebral venous pressure; however, it is not yet clear if this is the cause or a result of the intracranial hypertension [5].

Diagnosis of IIH is predominately a diagnosis of exclusion, with criteria that is described by the modified Dandy Criteria (Table 1). According to these criteria, those who 1) present with signs and symptoms indicating increased intracranial pressure, 2) lack localizing neurologic deficits except for sixth nerve palsies, 3) have increased CSF opening pressure with normal composition, 3) have no evidence of hydrocephalus or obstructions present, with no discernable cause for the intracranial hypertension, have IIH [6].

**Table 1:** Modified Dandy Criteria for the diagnosis of IIH. ICP: Intracranial pressure; CT: Computed tomography; MRI: Magnetic resonance imaging; CSF: Cerebrospinal fluid; IIH: Idiopathic intracranial hypertension.

Modified Dandy Criteria
Signs and symptoms of increased ICPs
Awake and alert patient
No localizing neurologic finding (except possible 6^th^ nerve palsy)
Normal CT/MRI findings
Increased CSF opening pressure (>20 cm H2O in non-obese patients, >25 cm H2O in obese patients
No other identifiable cause of increased ICPs

One of the Dandy criteria states that in IIH there are symptoms of increased intracranial pressure without evidence of hydrocephalus, which is an accumulation of fluid in the brain due to an imbalance in the production and absorption of CSF [7]. There are multiple forms of hydrocephalus based on the precipitating factor. The two main categories are obstructive and non-obstructive hydrocephalus. The former could be a result of a tumor, cyst, trauma, or multiple sclerosis plaques. The latter is generally a result of intracranial hemorrhage, subarachnoid hemorrhage or infection [8].

Obstructive hydrocephalus, also known as non-communicating hydrocephalus, is commonly a result of tumors or cysts in the pineal region, though it can arise from obstruction at any point in the pathway of normal CSF [7,8]. Obstructive hydrocephalus in adults can present with memory and gait disturbances, incontinence, blurred vision, seizures and headache. The treatment for this is dependent on the cause of the obstruction [9]. When possible, removal of the obstruction may result in normal CSF flow dynamics and resolution of the hydrocephalus. If removal of the obstruction is not possible or fails to alleviate the hydrocephalus, CSF diversion via ETV or shunting is often successful [10].

It has been observed that a majority of patients with IIH also have venous sinus stenosis [11]. It is currently unknown if the stenosis is a cause of the IIH or the result [12]. There are two distinct forms of transverse sinus stenosis, intrinsic and extrinsic [13]. There is some belief that these two morphologies may result from different etiologies. For example, the intrinsic stenosis might be a cause of IIH, while the extrinsic might be a result of the increased intracranial pressure [12]. Furthermore, an extrinsic stenosis will improve, when the intracranial pressure is reduced further supporting the belief that it is a consequence rather than a cause of the IIH [14].

While the initial cause of the stenosis is uncertain, it has been noted that correction of the stenosis can provide immediate improvement in the symptoms of IIH. Symptoms improve post stent placement in one sinus, regardless of whether the stenosis was intrinsic or extrinsic [13]. A study by Bussiere et al. reported that 82% of patients reported improvement of headache and 91% had improvement of their papilledema after stent placement [11]. Two recent meta-analyses reported even higher success rates with greater than 87% improvement in headaches and ~97% improvement in papilledema [15, 16]. Additionally, there was a statistically significant reduction in intracranial pressure within three months post stenting [14]. It has also been shown that there can be an immediate reduction of the intracranial pressure, with a mean decrease of 7.8 mm H2O following the stenting, an effect that was sustained beyond 24 hours [17]. Furthermore, the isolated sixth nerve palsy, which has been reported in IIH, has been reported to rapidly abate following stenting [18]. Venous sinus stenting is a newer treatment option for patients with IIH, with relatively few complications associated with it which include perforation of the vessel, stent migration and thrombosis [12,19]. However, in a large review by Starke et al. only 10 out of 185 patients had reported complications making stenting a safe and effective treatment for patients with IIH and venous sinus stenosis [20].

The patient discussed here presented with evidence of a mass in the pineal region and the patient’s symptomology was consistent with an indolent obstructive hydrocephalus. This presentation made the diagnosis of IIH difficult. However, when an appropriate treatment for obstructive hydrocephalus, an ETV in this case, had failed to yield improvement, the cause of her symptoms was understood to be from a separate pathology. Only after the possible obstruction had been eliminated and the patient’s symptoms failed to resolve could the diagnosis of IIH be yielded. The subsequent workup confirmed the diagnosis and with the proper treatment, adequate relief of symptoms was found. This case highlights the possible difficulty in diagnosing IIH. When evaluating a patient with the vague and often hard to describe complaints of headache and vision changes, the development of a broad differential is imperative. The initial evaluation should consist of a thorough history, including information of the duration, quality and inciting factors of the patient’s symptoms, as well as a thorough neurological exam that includes assessment of cranial nerves and visual fields. Ultimately, advanced imaging studies such as computed tomography and MR imaging will be required to evaluate for the presence of mass lesions or other processes contributing to the patient’s symptoms. In rare cases, as in this patient’s case, there may be “red herring” findings and appropriate clinical judgement is required to yield an accurate diagnosis. This case illustrates that IIH may present concurrently with mass lesions and obstructive hydrocephalus and clinicians should bear this possibility in mind when evaluating patients in the clinic.

## Conclusions

Idiopathic intracranial hypertension should be considered in the differential diagnosis for patients with signs or symptoms of elevated intracranial pressure. A thorough history and physical exam, as well as appropriate cranial imaging with dedicated venous imaging, can yield an accurate diagnosis. Evolving techniques to treat patients with IIH including venous sinus stenting can yield outstanding results in appropriately selected patients.
